# Supplementary material for: Selective sweep for an enhancer involucrin allele identifies skin barrier adaptation out of Africa
Source: Nat Commun. 2021 May 7;12:2557. doi: 10.1038/s41467-021-22821-w (PMC8105351; doi:10.1038/s41467-021-22821-w)
Supplement: Supplementary file 1 — Supplementary information [file 41467_2021_22821_MOESM1_ESM.pdf]

Supplementary Information

Selective sweep for an enhancer involucrin allele identifies skin barrier adaptation out of Africa

Mary Elizabeth Mathyer[1,2,3]*, Erin A. Brettmann[1,2,3]*, Alina D. Schmidt[1,2,3], Zane A. Goodwin[1,2,3], Inez Y. Oh[1,2,3], Ashley M. Quiggle[1,2,3], Eric Tycksen[4], Natasha Ramakrishnan[1,2,3], Scot A. Matkovich[2], Emma Guttman-Yassky[5], John R. Edwards[2], Cristina de Guzman Strong[1,2,3]¶

* equally contributing authors

¶ corresponding author

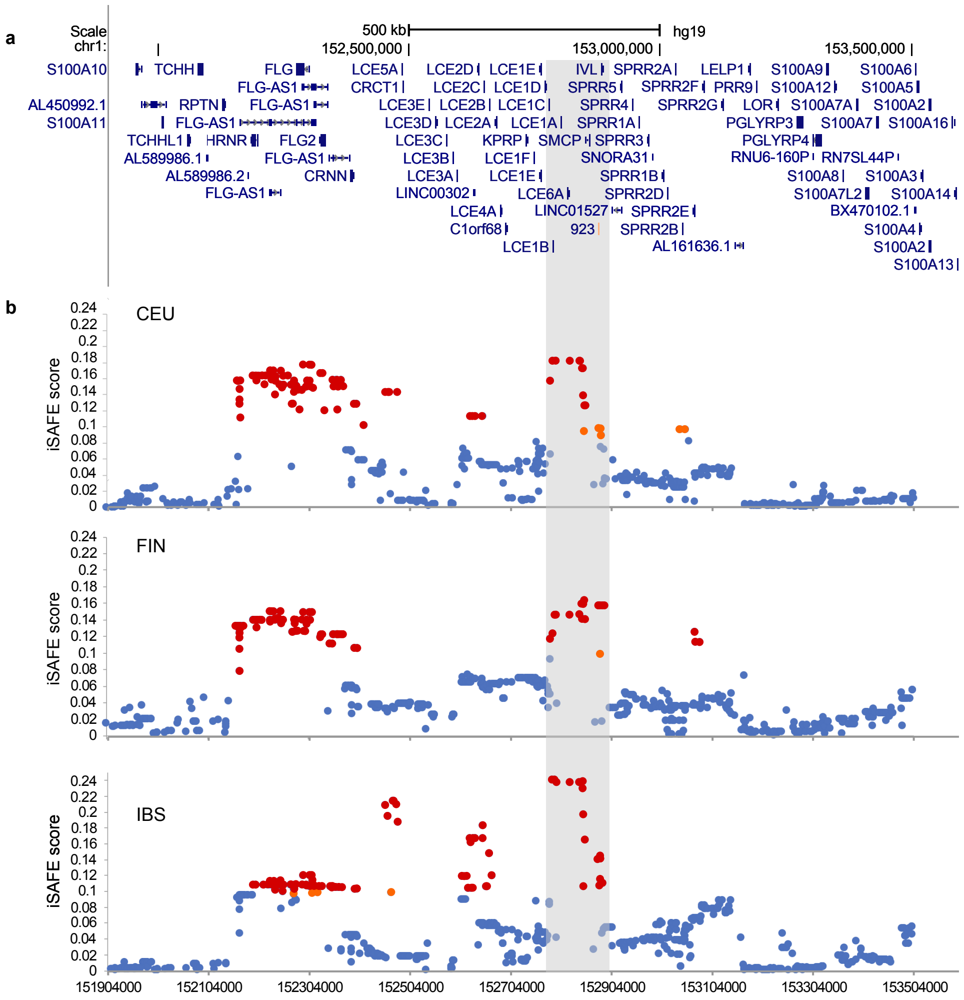

Supplementary Figure 1. Positive selection signals in the Epidermal Differentiation Complex in multiple European populations identify human evolution in the skin barrier. Positive selection within the **a**) EDC (hg19; chr1:151,904,000-153,593,700) was determined by **b**) SNPs with iSAFE scores > 0.10, all shown as red dots. Orange dots indicate SNPs 0.095<iSAFE score<0.10. The gray shaded region indicates the region of shared evidence of positive selection in CEU near *LCE1B-IVL*. iSAFE scores were calculated for 1KGP Phase 3 SNPs in CEU, FIN, and IBS.

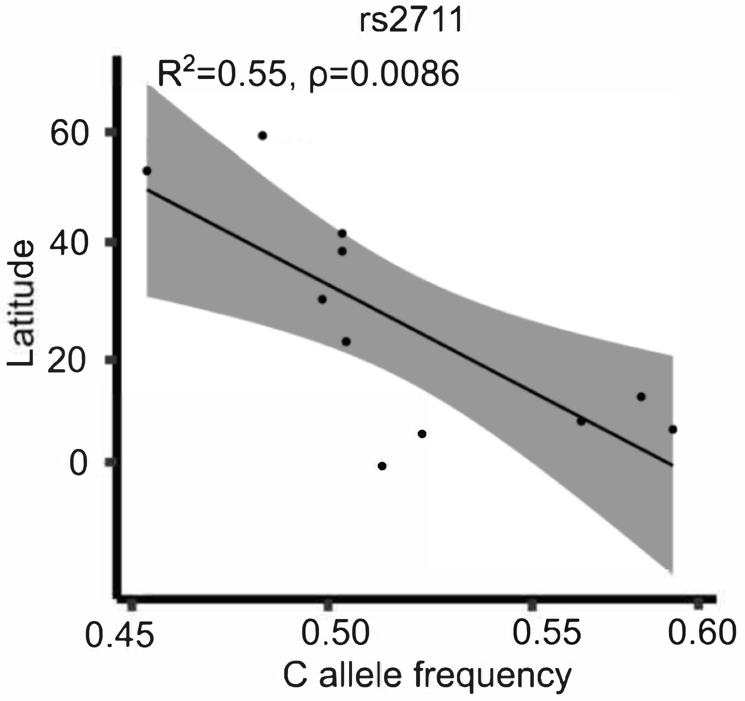

Supplementary Figure 2. No correlation between latitude and allele frequency for rs2711-C. The black line indicates a linear relationship (Pearson's correlation) between allele frequency and geographic latitude. Gray area surrounding the regression line represents the 95% confidence intervals for the group mean values of the latitude for each allele frequency. Two-sided t-test (11 degrees of freedom), 95% confidence intervals, $\rho = 0.0086$.

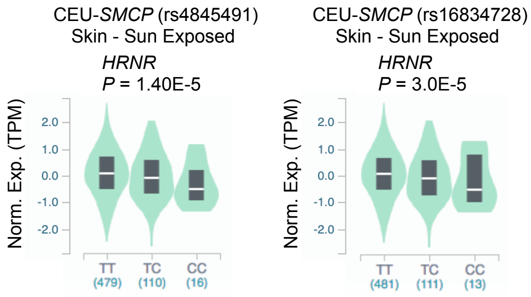

Supplementary Figure 3. Association of increased *HRNR* for CEU-*LCE1A-SMCP*. Violin plots for rs4845491-T and rs16834728-T in CEU-*LCE1A-SMCP*, eQTLs for increased *HRNR* expression in sun exposed skin (GTEx [V8]). Box in violin plot represents interquartile range with median (white line). Numbers in parentheses indicate number of individuals for each genotype. TPM, transcripts per million. Chi-Square *p*-value was calculated based on Mahalanobis distance and Bonferroni corrected.

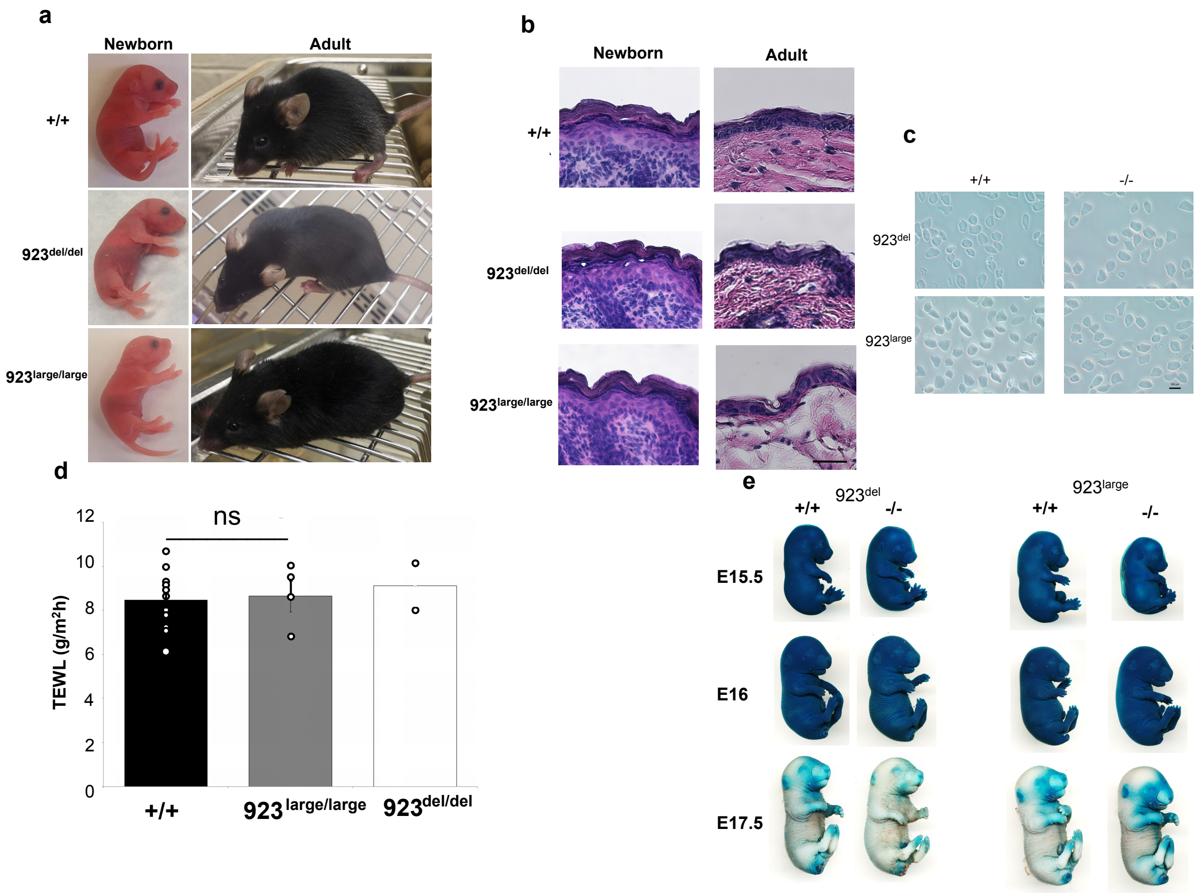

Supplementary Figure 4. Both 923 deletion mouse lines exhibit normal morphology under barrier-housed homeostatic conditions. a) Homozygous $923^{del/del}$ and $923^{large/large}$ mice are viable and appear normal in barrier-housed conditions at newborn or 8 weeks when compared to aged matched control WT (+/+) mice. **b**) H&E staining of WT, $923^{del/del}$, and $923^{large/large}$ epidermal sections from newborn and 8-week-old (adult) mice appear normal in 3 independent experiments per genotype. Scale bar, 150 μm. **c**) Normal cornified envelope morphology observed in homozygous $923^{del/del}$ and $923^{large/large}$ keratinocytes compared to WT littermates in 3 independent experiments per genotype. Similar quantities of angular and balloon shaped cornified envelopes with smooth edges were isolated from newborn skin of homozygous deletion and wildtype littermates of 923^{del} and 923^{large} mice. Scale bar, 500 μm. **d**) Normal inside-out skin barrier function in 923 deletion mice. Barrier function was measured by transepidermal water loss (TEWL) (+/+, n=16; $923^{large/large}$, n=4; $923^{del/del}$, n=2). Mean $\pm$ SEM. **e**) Normal patterning of skin barrier development in 923 deletion mice. The extent of skin barrier formation was assessed by an outside-in X-gal dye penetration assay. Blue stain indicates X-gal reactivity with endogenous β-galactosidase where the X-gal solution has penetrated the epidermis where the skin barrier has not formed.

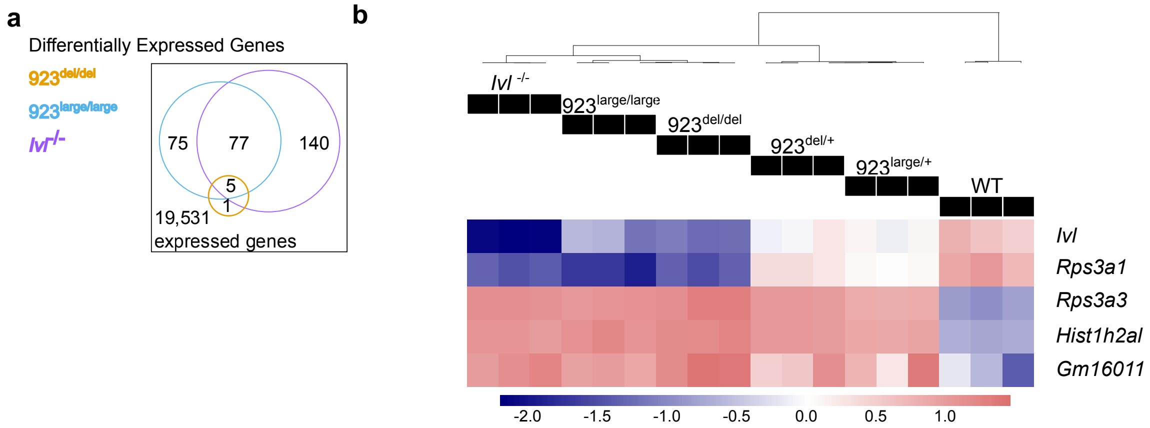

Supplementary Figure 5. RNA-seq comparison of differentially expressed genes in $923^{del/del}$, $923^{large/large}$ and *Ivl*-/- mice. a) Venn diagram of differentially expressed genes between $923^{del/del}$, $923^{large/large}$ and *Ivl*-/- compared to WT, respectively (FDR <0.05, log2(FC)>|2|). **b**) Heatmap of 5 differentially expressed genes in $923^{del/del}$, $923^{large/large}$ and *Ivl*-/- mice skin RNA-seq compared to WT (n=3/genotype).

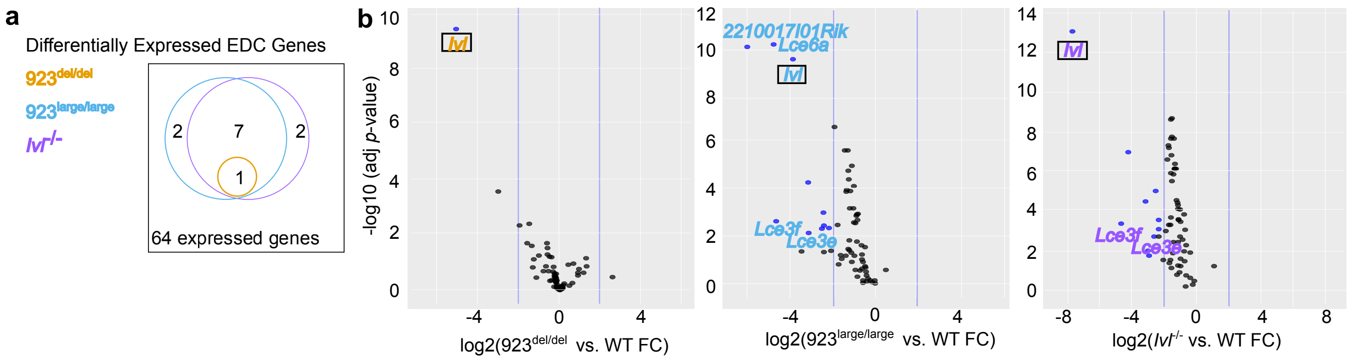

Supplementary Figure 6. RNA-seq comparison of differentially expressed genes in the EDC in 923$^{del/del}$, 923$^{large/large}$ and *Ivl*-/- mice. a) Venn diagram of differentially expressed EDC genes (FDR <0.05, log2(FC)>|2|) and **b**) volcano plots of EDC subset from differential gene expression analyses in 923$^{del/del}$, 923$^{large/large}$, and *Ivl*-/- newborn mouse skin each compared to WT (n=3/ genotype). Statistical analysis was done via Limma's generalized linear model moderated two-sided t-tests with 22 degrees of freedom and Benjamini-Hochberg false discovery rate (FDR) corrections.

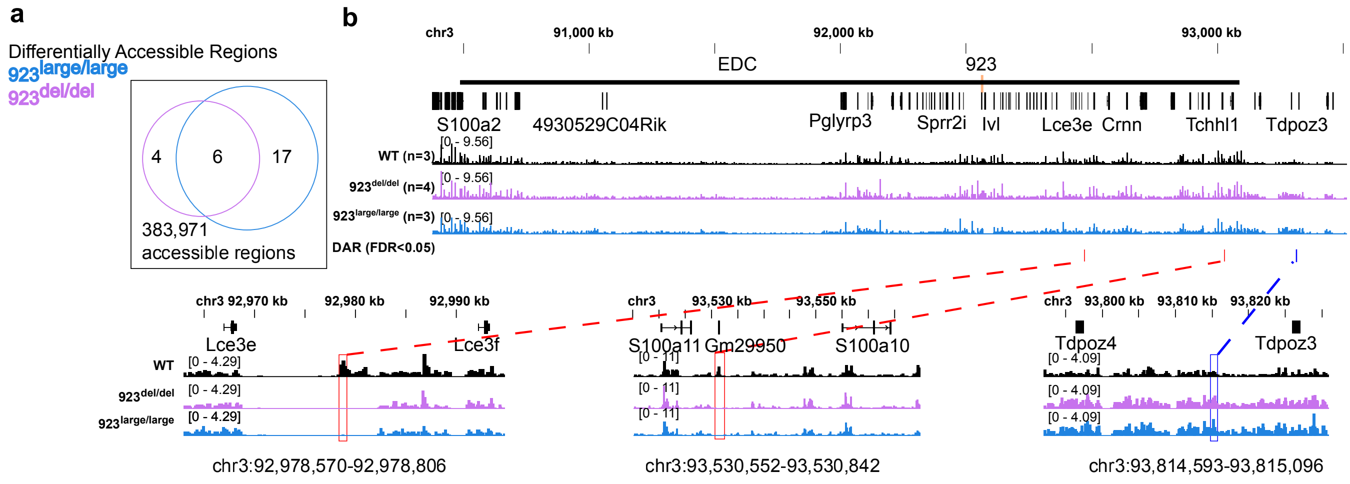

Supplementary Figure 7. Chromatin accessibility is altered in and around the EDC in 923$^{del/del}$ and 923$^{large/large}$ newborn epidermis. a) Venn diagram (FDR <0.05) of all differentially accessible regions (DARs) using comparative ATAC-seq in 923$^{del/del}$ and 923$^{large/large}$ newborn epidermis that were each compared to WT with **b**) an enrichment of shared DARs in the EDC. Peaks shown as fold change signal per genotype (WT, n=3; 923$^{del/del}$, n=4; 923$^{large/large}$, n=3) with shared less accessible (red) and more accessible (blue) DARs indicated in the 923 deletion lines compared to WT.

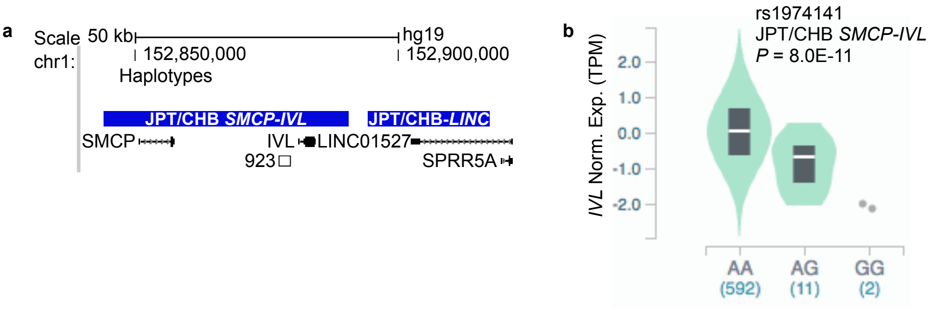

Supplementary Figure 8. JPT/CHB-*SMCP-IVL* is associated with relatively decreased *IVL* expression. a) Phasing reveals JPT/CHB-*SMCP-IVL* haplotype (blue bar) based on SNPs in linkage disequilibrium (r^2>0.8) with rs6668295. **b**) Violin plots for rs1974141-G in JPT/CHB-*SMCP-IVL*, eQTL for decreased *IVL* expression in sun exposed skin (GTEx [V8]). Box in violin plot represents interquartile range with median (white line). Numbers in parentheses indicate number of individuals for each genotype. TPM, transcripts per million. Chi-Square *p*-value was calculated based on Mahalanobis distance and Bonferroni corrected.

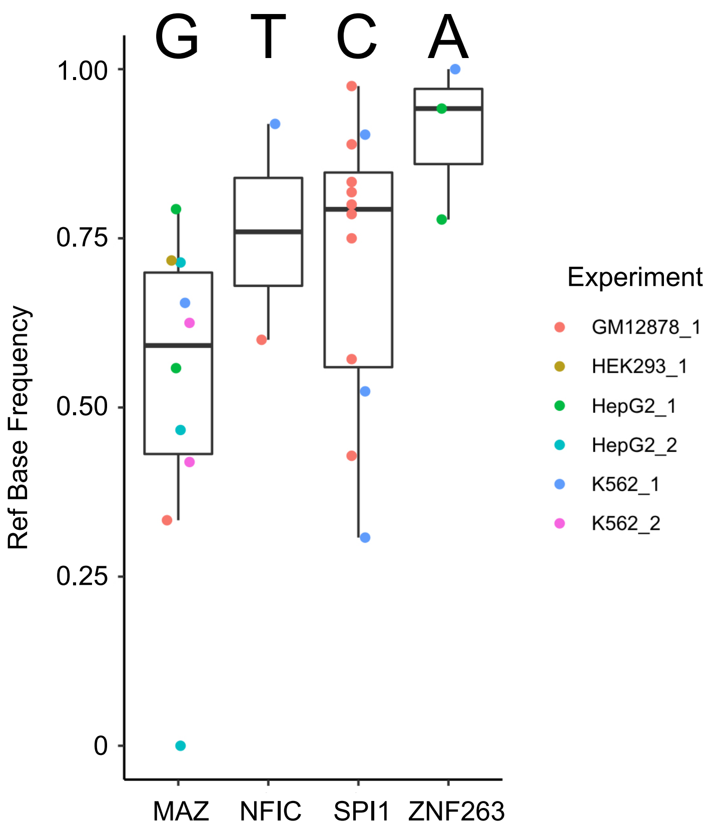

Supplementary Figure 9. Preferential binding in vivo for transcription factor at predicted allele. MAZ (G), NFIC (T), and SPI1 (C), and ZNF263 (A) at polymorphic sites for ENCODE ChIP-seq datasets. Center line, median; box limits, upper and lower quartiles; whiskers, 1.5X interquartile range or largest or smallest point. The following independent cell experiments were used for each transcription factor: MAZ (n=9), NFIC (n=2), SPI1 (n=12), and ZNF263 (n=3).

Supplementary Table 1. CEU-IVL SNPs identified by CMS/iSAFE and iSAFE alone are also GTEx eQTLs (V8) found in sun exposed and not sun exposed skin. Chi-Square *P*-value was calculated based on Mahalanobis distance and Bonferroni corrected. Normalized effect size = NES.

					Skin - Sun Exposed						*Skin - Not Sun Exposed*					
rsID	*Chr*	*Position (hg19)*	*Ref (CEU)*	*Alt*	*IVL*		*LCE1E*		*LCE1D*		*IVL*		*LCE1E*		*LCE1D*	
					NES	*P value*	*NES*	*P value*	*NES*	*P value*	*NES*	*P value*	*NES*	*P value*	*NES*	*P value*
rs4845327	1	152879512	G	T	-0.28	4.0E-15	-0.23	2.8E-05	None	None	-0.26	2.3E-09	-0.29	1.5E-06	-0.33	2.4E-11
rs1854779	1	152880672	T	C	-0.29	6.8E-16	-0.24	5.7E-06	-0.23	2.4E-04	-0.26	9.0E-10	-0.32	1.1E-07	-0.35	4.5E-07
rs7539232	1	152881802	G	A	-0.29	6.8E-16	-0.24	5.7E-06	-0.23	2.4E-04	-0.26	9.0E-10	-0.32	1.1E-07	-0.35	4.5E-07
rs11205132	1	152882135	A	G	-0.29	6.8E-16	-0.24	5.7E-06	-0.23	2.4E-04	-0.26	9.0E-10	-0.32	1.1E-07	-0.35	4.5E-07
rs2229496	1	152882610	A	G	-0.29	6.8E-16	-0.24	5.7E-06	-0.23	2.4E-04	-0.26	9.0E-10	-0.32	1.1E-07	-0.35	4.5E-07
rs7535306	1	152883680	A	G	-0.29	6.8E-16	-0.24	5.7E-06	-0.23	2.4E-04	-0.26	9.0E-10	-0.32	1.1E-07	-0.35	4.5E-07
rs7545520	1	152883711	G	C	-0.29	6.8E-16	-0.24	5.7E-06	-0.23	2.4E-04	-0.26	9.0E-10	-0.32	1.1E-07	-0.35	4.5E-07

Supplementary Table 2. Offspring genotype distribution from heterozygous 923^{del} as well as 923^{large} parental crosses.
Chi-squared test calculations included.

923^{del} Het Intercrosses

Genotype	*Observed*	*Expected*		
+/+	22	18.75		
+/-	40	37.5		
-/-	13	18.75	Chi-sqr test =	0.287461

923^{large} Het Intercrosses

Genotype	*Observed*	*Expected*		
+/+	21	19.25		
+/-	38	38.5		
-/-	18	19.25	Chi-sqr test =	0.883931

Supplementary Table 3. Ranked list of differentially expressed genes between 923$^{del/del}$ and WT mice whole skin from RNA-seq. List ranked by log2FC. Statistical analysis using Limma's generalized linear model moderated two-sided t-tests with 22 degrees of freedom and FDR (Benjamini-Hochberg, adj. *p* val) <0.05 and logFC <|2| cutoffs were used.

Feature_ID	*entrezgene*	*external_gene_name*	*gene_biotype*	*description*	*logFC*	*adj. p val*
ENSMUSG00000049128	16447	*Ivl*	protein_coding	involucrin [Source:MGI Symbol;Acc:MGI:96626]	-5.119	1.98E-06
ENSMUSG00000028081	20091	*Rps3a1*	protein_coding	ribosomal protein S3A1 [Source:MGI Symbol;Acc:MGI:1202063]	-4.879	9.60E-14
ENSMUSG00000081355	NA	*Gm15264*	unprocessed_pseudogene	predicted gene 15264 [Source:MGI Symbol;Acc:MGI:3705845]	2.482	3.18E-03
ENSMUSG00000081303	NA	*Gm16011*	processed_pseudogene	predicted gene 16011 [Source:MGI Symbol;Acc:MGI:3801796]	4.155	3.46E-02
ENSMUSG00000091383	NA	*Hist1h2al*	processed_pseudogene	histone cluster 1, H2al [Source:MGI Symbol;Acc:MGI:3646032]	7.650	8.65E-10
ENSMUSG00000059751	NA	*Rps3a3*	processed_pseudogene	ribosomal protein S3A3 [Source:MGI Symbol;Acc:MGI:3643406]	8.451	5.17E-11

Supplementary Table 4. Ranked list of differentially expressed genes between 923del/+ and WT mice whole skin from RNA-seq. List ranked by log2FC. Statistical analysis using Limma's generalized linear model moderated two-sided t-tests with 22 degrees of freedom and FDR (Benjamini-Hochberg, adj. *p* val) <0.05 and logFC <|2| cutoffs were used.

Feature_ID	*entrezgene*	*external_gene_name*	*gene_biotype*	*description*	*logFC*	*adj. p*
ENSMUSG00000040852	213556	*Plekhh2*	protein_coding	pleckstrin homology domain containing, family H (with MyTH4 domain) member 2 [Source:MGI Symbol;Acc:MGI:2146813]	-4.544	1.78E-02
ENSMUSG00000078122	NA	*F630028O10Rik*	antisense	RIKEN cDNA F630028O10 gene [Source:MGI Symbol;Acc:MGI:3641813]	-2.150	4.04E-02
ENSMUSG00000111912	NA	*Gm48521*	lincRNA	predicted gene, 48521 [Source:MGI Symbol;Acc:MGI:6098057]	-2.142	3.65E-02
ENSMUSG00000074634	633640	*Tmem267*	protein_coding	transmembrane protein 267 [Source:MGI Symbol;Acc:MGI:3648543]	-2.009	2.05E-02
ENSMUSG00000049103	12772	*Ccr2*	protein_coding	chemokine (C-C motif) receptor 2 [Source:MGI Symbol;Acc:MGI:106185]	-2.000	3.65E-02
ENSMUSG00000049128	16447	*Ivl*	protein_coding	involucrin [Source:MGI Symbol;Acc:MGI:96626]	-1.827	2.59E-03
ENSMUSG00000039252	246316	*Lgi2*	protein_coding	leucine-rich repeat LGI family, member 2 [Source:MGI Symbol;Acc:MGI:2180196]	2.104	3.85E-02
ENSMUSG00000035769	102448	*Xylb*	protein_coding	xylulokinase homolog (H. influenzae) [Source:MGI Symbol;Acc:MGI:2142985]	2.602	3.67E-02
ENSMUSG00000035184	629059	*Fam124a*	protein_coding	family with sequence similarity 124, member A [Source:MGI Symbol;Acc:MGI:3645930]	2.621	4.72E-02
ENSMUSG00000040289	15213	*Hey1*	protein_coding	hairy/enhancer-of-split related with YRPW motif 1 [Source:MGI Symbol;Acc:MGI:1341800]	2.711	1.78E-02
ENSMUSG00000016346	16536	*Kcnq2*	protein_coding	potassium voltage-gated channel, subfamily Q, member 2 [Source:MGI Symbol;Acc:MGI:1309503]	2.924	3.21E-02
ENSMUSG00000066191	75691	*Anks6*	protein_coding	ankyrin repeat and sterile alpha motif domain containing 6 [Source:MGI Symbol;Acc:MGI:1922941]	2.976	1.20E-02
ENSMUSG00000039137	73750	*Whrn*	protein_coding	whirlin [Source:MGI Symbol;Acc:MGI:2682003]	3.023	6.02E-04
ENSMUSG00000069227	26913	*Gprin1*	protein_coding	G protein-regulated inducer of neurite outgrowth 1 [Source:MGI Symbol;Acc:MGI:1349455]	3.505	3.65E-02
ENSMUSG00000006538	16147	*Ihh*	protein_coding	Indian hedgehog [Source:MGI Symbol;Acc:MGI:96533]	3.882	3.02E-02
ENSMUSG00000027517	70065	*Ankrd60*	protein_coding	ankyrin repeat domain 60 [Source:MGI Symbol;Acc:MGI:1917315]	3.939	4.36E-02
ENSMUSG00000092675	NA	*Gm25262*	miRNA	predicted gene, 25262 [Source:MGI Symbol;Acc:MGI:5455039]	3.974	1.76E-02
ENSMUSG00000098973	NA	*Mir6236*	miRNA	microRNA 6236 [Source:MGI Symbol;Acc:MGI:5530929]	4.119	4.04E-02
ENSMUSG00000033948	74464	*Zswim5*	protein_coding	zinc finger SWIM-type containing 5 [Source:MGI Symbol;Acc:MGI:1921714]	4.237	1.76E-02
ENSMUSG00000076258	NA	*Gm23935*	miRNA	predicted gene, 23935 [Source:MGI Symbol;Acc:MGI:5453712]	4.258	1.15E-02
ENSMUSG00000092909	NA	*Gm25732*	miRNA	predicted gene, 25732 [Source:MGI Symbol;Acc:MGI:5455509]	4.613	2.32E-02
ENSMUSG00000112365	NA	*Gm49782*	lincRNA	predicted gene, 49782 [Source:MGI Symbol;Acc:MGI:6215301]	4.871	3.21E-02
ENSMUSG00000044518	30923	*Foxe3*	protein_coding	forkhead box E3 [Source:MGI Symbol;Acc:MGI:1353569]	5.296	3.21E-02
ENSMUSG00000091383	NA	*Hist1h2al*	processed_pseudogene	histone cluster 1, H2al [Source:MGI Symbol;Acc:MGI:3646032]	7.024	7.31E-09
ENSMUSG00000059751	NA	*Rps3a3*	processed_pseudogene	ribosomal protein S3A3 [Source:MGI Symbol;Acc:MGI:3643406]	7.605	9.56E-10

Supplementary Table 5. Ranked list of differentially accessible regions between 923$^{del/del}$ and WT mice epidermis from ATAC-seq. List ranked by FC. FDR <0.05 and FC <|2| cutoffs used.

Chromosome	*start*	*end*	*Fold*	*FDR*
chrX	169993996	169994243	-4.64	2.21E-02
chrX	169996914	169998486	-3.70	2.66E-02
chr3	93176562	93176984	-2.76	4.62E-02
chr3	93814593	93815096	-2.56	8.59E-03
chr3	79242295	79242558	2.89	4.62E-02
chrX	50591524	50591728	3.78	3.78E-02
chrX	50611755	50611998	4.19	3.78E-02
chr3	78966708	78967396	4.34	3.81E-07
chr3	92978570	92978806	4.39	1.04E-03
chr3	93530552	93530842	4.82	3.81E-07

Supplementary Table 6. Ranked list of differentially accessible regions between 923$^{large/large}$ and WT mice epidermis from ATAC-seq. List ranked by FC. FDR <0.05 and FC <|2| cutoffs used.

Chromosome	*start*	*end*	*Fold*	*FDR*
chrX	169993996	169994243	-5.41	9.91E-05
chrX	169996914	169998486	-3.83	4.87E-04
chr8	119234019	119234204	-3.66	2.97E-02
chr7	118642175	118642353	-3.63	4.64E-02
chr12	118918105	118918297	-3.59	4.41E-02
chr8	86904158	86904360	-3.47	2.43E-02
chr15	88995738	88995926	-3.45	1.59E-02
chr14	37306986	37307136	-3.43	4.31E-02
chr14	7972236	7972440	-3.41	7.92E-03
chr5	38901570	38901772	-3.23	3.23E-02
chr3	93780248	93780977	-2.73	7.28E-03
chr3	93814593	93815096	-2.53	7.28E-03
chr13	21172295	21172487	2.99	4.31E-02
chr3	92978570	92978806	3.64	4.31E-02
chr3	92586198	92586460	3.64	4.71E-02
chrX	52243896	52244124	3.72	1.04E-02
chr3	78966708	78967396	3.99	1.19E-04
chr5	123127103	123127309	4.00	1.86E-02
chr3	92579546	92579913	4.54	4.87E-04
chr3	92583166	92583453	4.61	9.91E-05
chr3	93530552	93530842	4.84	8.89E-06
chr3	92609764	92610128	5.50	5.05E-07
chr1	24613142	24615948	5.69	1.04E-02

Supplementary Table 7. SNPs in JPT/CHB allele are GTEx eQTLs for *IVL* (V8) in sun exposed and not sun exposed skin. Chi-Square *P*-value was calculated based on Mahalanobis distance and Bonferroni corrected. Normalized effect size = NES.

rsID	*Chr*	*Position (hg38)*	*Ref (CEU)*	*Alt (JPT/CHB cloned allele)*	*Skin - Sun Exposed IVL*		*Skin - Not Sun Exposed IVL*	
					NES	*P Value*	*NES*	*P Value*
rs1974141	1	152907036	A	G	-0.85	8.00E-11	-1.0	3.3E-09
rs12036697	1	152906433	A	G	-0.85	8.00E-11	-1.0	3.3E-09
rs16834746	1	152906620	T	C	-0.85	8.00E-11	-1.0	3.3E-09
rs4845327	1	152907036	G	T	-0.28	4.00E-15	-0.26	2.30E-09
rs1854779	1	152908196	T	C	-0.29	6.80E-16	-0.25	9.00E-09
rs16834751	1	152908735	A	C	-0.43	9.90E-07	-0.51	3.90E-06
rs4523473	1	152908954	T	C	-0.28	1.90E-15	-0.24	2.90E-08
rs11205130	1	152909173	T	C	-0.85	8.00E-11	-1.0	3.30E-09
rs11205131	1	152909202	G	A	-0.53	1.10E-07	-0.52	2.00E-05
rs7528862	1	152909213	A	G	-0.29	6.80E-16	-0.26	9.00E-10
rs7517189	1	152909270	C	G	-0.29	6.80E-16	-0.26	9.00E-10
rs7539232	1	152909326	G	A	-0.29	6.80E-16	-0.26	9.00E-10
rs11205132	1	152909659	A	G	-0.29	6.80E-16	-0.26	9.00E-10

Supplementary Table 8. Transcription factor binding predictions for *IVL* eQTLs (V8).

IVL eQTL	*Regulatory Element*	*hg38 position Chr 1*	*Reference (CEU; major)*	*Alternate (Minor)*	*Conserved in Mouse*	*Effect size for minor (sun-exposed skin GTEX v8)*	*Effect size for minor (Not sun-exposed skin GTEX v8)*	*TFBS (Ref)*	*TFBS (Alt)*
rs1974141	enhancer	152905746	A	G	No	-0.85	-1.0	ZNF263	MAZ
rs12036697	enhancer	152906433	A	G	No	-0.85	-1.0	AP-1	AP-1
rs16834746	enhancer	152906620	T	C	Yes (T)	-0.85	-1.0	None	None
rs4845327	enhancer	152907036	G	T	Yes (T)	-0.28	-0.26	IRF1	SOX10
rs1854779	promoter	152908196	T	C	Yes (T)	-0.29	-0.26	NFIC	SPI1
rs16834751	intron	152908735	A	C	No	-0.43	-0.51	None	None
rs4523473	intron	152908954	T	C	No	-0.28	-0.24	CTCF	CTCF
rs11205130	intron	152909173	T	C	Yes (T)	-0.85	-1.0	TFAP2A	TFAP2A
rs11205131	intron	152909202	G	A	No	-0.53	-0.52	MZF1	MZF1
rs7528862	intron	152909213	A	G	Yes (A)	-0.29	-0.26	IRF1	IRF1
rs7517189	intron	152909270	C	G	Yes (T)	-0.29	-0.26	REL	GATA3
rs7539232	intron	152909326	G	A	Yes (C)	-0.29	-0.26	None	None
rs11205132	intron	152909659	A	G	Yes (G)	-0.29	-0.26	FOXC1	FOXC1

Supplementary Table 9. Preferential binding *in vivo* for ZNF263 (A), MAZ (G), NFIC (T), and SPI1 (C) to predicted alleles at polymorphic sites in ENCODE ChIP-seq datasets. FIMO computes a log-likelihood ratio score for each motif at each position and converts these scores to *P*-values using dynamic programming (Supplementary Ref.1).

Cell_line	*Replicate*	*TF*	*P value*	*chrom*	*Motif Position (hg38)*	*SNP position (hg38)*	*strand*	*Ref*	*Alt*	*Rel_pos in Motif*	*Coverage*	*A*	*C*	*G*	*T*	*Dist_to_center*	*Ref_cnt*	*Alt_cnt*	*Ref_freq*
HepG2	1	ZNF263	1.00E-05	chr1	44031571	44031577	-	T	C	6	106	0	6	3	97	20.5	97	6	0.942
HepG2	1	ZNF263	1.00E-05	chr9	136251492	136251499	+	A	G	7	9	7	0	2	0	36	7	2	0.778
K562	1	ZNF263	1.00E-05	chr1	44031571	44031577	-	T	C	6	5	0	0	0	5	22.5	5	0	1.000
GM12878	1	MAZ	1.00E-05	chr11	20610306	20610313	-	G	A	7	12	8	0	4	0	25	4	8	0.333
HEK293	1	MAZ	1.00E-05	chr1	11934650	11934657	-	G	A	7	145	41	0	104	0	27.5	104	41	0.717
HepG2	1	MAZ	1.00E-05	chr5	150449741	150449748	-	G	A	7	43	19	0	24	0	1	24	19	0.558
HepG2	1	MAZ	1.00E-05	chr20	62144533	62144538	+	C	T	5	29	0	23	0	6	0	23	6	0.793
HepG2	2	MAZ	1.00E-05	chr3	187740026	187740033	-	G	A	7	7	7	0	0	0	13	0	7	0.000
HepG2	2	MAZ	1.00E-05	chr5	150449741	150449748	-	G	A	7	14	4	0	10	0	5	10	4	0.714
HepG2	2	MAZ	1.00E-05	chr9	95876019	95876024	+	C	T	5	15	0	7	0	8	27	7	8	0.467
K562	1	MAZ	1.00E-05	chr11	20610306	20610313	-	G	A	7	55	19	0	36	0	15	36	19	0.655
K562	2	MAZ	1.00E-05	chr11	20610306	20610313	-	G	A	7	48	18	0	30	0	20	30	18	0.625
K562	2	MAZ	1.00E-05	chr15	40565034	40565041	-	G	A	7	31	18	0	13	0	50	13	18	0.419
GM12878	1	NFIC	5.00E-04	chr1	247407515	247407517	+	T	C	2	15	0	6	0	9	0	9	6	0.600
K562	1	NFIC	5.00E-04	chr8	3183715	3183717	+	T	C	2	173	0	14	0	159	7	159	14	0.919
GM12878	1	SPI1	5.00E-04	chr14	89882066	89882071	-	C	T	5	27	0	24	0	3	17.5	24	3	0.889
GM12878	1	SPI1	5.00E-04	chr19	47435401	47435406	-	C	T	5	40	0	39	0	1	4	39	1	0.975
GM12878	1	SPI1	5.00E-04	chr2	11754812	11754817	-	C	T	5	21	0	9	0	12	6.5	9	12	0.429
GM12878	1	SPI1	5.00E-04	chr20	17961089	17961094	-	C	T	5	14	0	11	0	3	22	11	3	0.786
GM12878	1	SPI1	5.00E-04	chr8	41713910	41713915	-	C	T	5	7	0	4	0	3	48	4	3	0.571
GM12878	1	SPI1	5.00E-04	chr12	120698927	120698929	+	G	A	2	10	2	0	8	0	11	8	2	0.800
GM12878	1	SPI1	5.00E-04	chr18	2641047	2641049	+	G	A	2	11	2	0	9	0	13	9	2	0.818
GM12878	1	SPI1	5.00E-04	chr5	109410410	109410412	+	G	A	2	6	1	0	5	0	6	5	1	0.833
GM12878	1	SPI1	5.00E-04	chr7	24255372	24255374	+	G	A	2	12	3	0	9	0	34	9	3	0.750
K562	1	SPI1	5.00E-04	chr11	124339876	124339881	-	C	T	5	31	0	28	0	3	12	28	3	0.903
K562	1	SPI1	5.00E-04	chr7	27400396	27400401	-	C	T	5	21	0	11	0	10	12	11	10	0.524
K562	1	SPI1	5.00E-04	chr18	46711454	46711456	+	G	A	2	26	18	0	8	0	45	8	18	0.308

Supplementary Table 10. CRISPR/Cas9 editing strategy reagents and mouse allele sequences.

a. Small Guide RNA (sgRNA) targeting sequences

Upstream 5' sgRNA	5' - GAATACATCCCAGGAACAT -3'
Downstream 3' sgRNA	5' - CAGTAAGCTAGCGCTAGAC -3'

b. ssODN sequences: SphI restriction site, LoxP site, HindIII restriction site, **Homology arms**

Upstream 5' ssODN

5’-
AGAAGTTTTTCAGTTCCCCATAGTTGTCCTGAGGAGCATATAATCTTTGTCTTAAGCA
GATTTGTTTACAATAATTCCCTATAACTTCGTATAGCATACATTATACGAAGTTATGCAT
GC**TTTAAAGAGATAGAGGACTGACATGACCCTCTGTCCTCTAAAACAAGTTTGCCA**
GGATTTCTCCATTCCCAGAGCCATGA-3’

Downstream 3'ssODN

5' -
TCTCTGTTGTTAGAGTCCATCTCCTACACCGATAGAGACTGATTCTGAAAAAAAAGG
AAGCTCCCACTGTCCAAGTTCTAAAGCTTATAACTTCGTATAGCATACATTATACGAAG
TTATT**GGAAACCAGACACCCTGGCTGCTGCTCTGAAGGCAACTCTTCCCTATCAGGC**
TCCTTAATAGGATTTGATCAGTGTGAC-3’

c. 923 WT

TCTTTAGTGCTCAGTTAACAGCTTATTTTATGGAGTTCATCATTAACACTTTTTTATGAGA
TCATACAAAATAATATAGTAAAATAATGGAAAGATAAAACTCATTTCTAATTAGTCTTG**AG**
AAGTTTTTCAGTTCCCCATAGTTGTCCTGAGGAGCATATAATCTTTGTCTTAAGCAGATTTG
TTTACAATAATTCCCTATGTTCCTGGGATGTATTC**TTTAAAGAGATAGAGGACTGACATGAC**
CCTCTGTCCTCTAAAACAAGTTTGCCAGGATTTCTCCATTCCCAGAGCCATGAGGCATCCT
GAACACTACTCTGAACTATATTTCTTTCTTCCTTTCTTTCTTCCTTCCTTCCTTCCTTCCT
TCCTTCCTTCCTTCCTTCCTTCCTTCCTTCCTTTCTTCCTTCCTTCTTTTCTTTTTCTTTCTTTC
TTTCTTTCTTTCTTTCTTTCTTTCTTTCTTTTTCTCTTTTTCTTTCCTTTTTTTGCTTTTATTTATT
TTTTTTCTTTTTCTATTAGATATTTTCTTCATTTACATTTCAAATGTTATCCCCAAGCCCCCTATA
CCCTCCCCCAGTCCTGATCCCCAACCCACCCACACCCACTTCCTGGCCCTGGCACTACTTTC
TATACCAAAGAAAGCATTCCCCACCCCACCAGAAGAAGTAAGCAAGCATTCTCACATGAGCAC
TTATGCTCCACTTCTGACTTCACATGGGAAGAATCTGACTCTCCTCAACCTGTGACAGTGCCA
GGGCAGCAGACTGGTCAAAAAGTCACACTGGTCTTATGGGTTCCCAGAGGCTCAGTATCTG
CTCAATCTGTTTCCCACCAGCTGATTCAGAGTATGATAAGAATTCAGAAATGATACTGTGTGT
GCGTGAGTGTTTGAGCACTGGGAAAAGCTAAGGTGTGGGAATGAGGGCATAGGATAGAGCC
CAGAAACCTGTGTGTAGTTGAAGGAGGGGTTGAAGAAGCTCCAGACTTCTAATGCTCAAAGG
TCACATATTTTGCCCTAGGATTATCCCACTTAGCGACTGGGAATGCATGTCAATTTGGCATTT
TTTTTTCAGTGTGCTGTGTGACTGACTTTATAAGTCTCAGATCCTTGTGATGAATCCAAGAAA
CTATGCAATGCAAATTATACAAATTCTCCAGTGTAATGAAGGTAACCTTTCCCATAACCCATGA
AGAGGCCTTGACCCAGCTCGGCCTCAGTGTTTAGGAGGATAAGAGAAGGTGAAGGGATGAA
TATGACCAGAATGTGTGAAATTGGCAGAGAATGAATTATTTCTGAAAACTTGCCTTTGAAGAG
TTTAGAGTGCTGCAGCTTCTTCAGAGAACATCA**TCTCTGTTGTTAGAGTCCATCTCCTACAC**
CGATAGAGACTGATTCTGAAAAAAAAGGAAGCTCCCACTGTCCAAGTTCTACAGTAAGCTA
GCGCTAGAC**TGGAAACCAGACACCCTGGCTGCTGCTCTGAAGGCAACTCTTCCCTATCAG**
GCTCCTTAATAGGATTTGATCAGTGTGACAGGTTTCACTACATGACTACAGAGACATCCTCT
AAGTCCAATAAGTTCCTGTGAGAATTTGGTGAGGCA

d. 923del

TCTTTAGTGCTCAGTTAACAGCTTATTTTATGGAGTTCATCATTAACACTTTTTTATGAGA
TCATACAAAATAATATAGTAAAATAATGGAAAGATAAAACTCATTTCTAATTAGTCTTG**AG**
AAGTTTTTCAGTTCCCCATAGTTGTCCTGAGGAGCATATAATCTTTGTCTTAAGCAGA
TTTGTTTACAATAATTCCCACTGTCCACTAAAGCTTATAACTTCGTATAGCATACATTAT
ACGAAGTTAT**TGGAAACCAGACACCCTGGCTGCTGCTCTGAAGGCAACTCTTCCCTA**
TCAGGCTCCTTAATAGGATTTGATCAGTGTGACAGGTTTCACTACATGACTACAGAGA
CATCCTCTAAGTCCAATAAGTTCCTGTGAGAATTTGGTGAGGCAA

e. 923large

TCCTCTGAATGCCCTAACTATCAGATTGTNTTCAGCTTTAATTAACTAATAAATTTTAGTT
ATTCTATCTATATTTATTTCATATTATTTATCTGTCTTCCACTGAAAAACAAGTTATATTTT
GAGAGAAATATTCTGGGTGTGCTTTCCATTGTCTCAAGGACCTATCAAAGTCACTCCAT
ACACTAAAACACTATCAGTATTAATTAAAAGAATAAATGACAGCAAATCTCATACCTACA
GACAACAACTTCCTAATATTTTAATGTCAAACAATCTTCATGTGTTTGAAAATGTGTGTG
CTAGGAAAATAAAGCTGAATTGTGGCTTATTTTTGTCTTTAGTGCTCAGTTAACAGCTTAT
TTTATGGAGTTCATCATTAACACTTTTTTATGAGATCATACAAAATAATATAGTAAAATAAT
GGAAAGATAAAACTCATTTCTAATTAGTCTTG**AGAAGTTTTTCAATTTCCCATAGTTGTC**
CTGAGGAGCATATAATCTTTGTCTTAAGCAGATTTGTTTACAATAATTCCCTATAACTT
CGTATAGCATACATTATACGAAGTTATGCATGC**TTTAAAGAGATAGAGGACTGA**TCAT
GGACTTGGTAAATAGCCATATAAAATAGGAGCAGGTGGAAAAAAACATTTTCATTT**CTG**
ATTCTGAAAAAAAAGGAAGCTCCCACTGTCCAAGTTCTAAAGCTTATAACTTCGTATA
GCATACATTATACGAAGTTAT**TGGAAACCAGACACCCTGGCTGCTGCTCTGAAGGCA**
ACTCTTCCCTATCAGGCTCCTCAATAGGTGTTCTACATGAATGTATTGCTATGAAGCTA
CAGAGAACTGAAATACAAATTCCCAGAAATCTGTCCCTGAGAGGAGAAGAACCCACTT
GAGGGTCCTCTGCACTTCTGATCAGGGTCTCAAGAACTCACAGAAATCACAGTTATGC
ACCATGATCAATTTTATTGTTGTTGAACTGAAAGGTAGGCTAAAGAAAGAAACAAGAAAT
GTTTTTCTAGCCAAGAGAGGTGGAGGG

Supplementary Table 11. Primers and Allele-Specific Amplicons. Amplicon sequences with SNPs bolded and primers underlined.

Primer Name	*Primer Sequence*	*Experiment*
Ivl For	TGGGTCAGTCACTTAAGCAAGA	Allele-specific expression of *Ivl* in mouse
Ivl Rev	TTCTGCTGCTGCTTCTCTGT	
2210017I01Rik For	GGTCCCCAGGTTCCTACTTC	Allele-specific expression of *2210017I01Rik* in mouse
2210017I01Rik Rev	TCAAAGCTTATCCTGGGCCA	
Lce6a For	TCCAGAACACTGTCAGCCAT	Allele-specific expression of *Lce6a* in mouse
Lce6a Rev	GCACCATGATCAATTTTATTGTTG	
PCR primer1	AATGATACGGCGACCACCGAGATCTACACTCTTTCCCTACACGACGCTCTTCCGATCT	RNA-seq
PCR primer2	GTGACTGGAGTTCAGACGTGTGCTCTTCCGATCT	
Index Primer1	CAAGCAGAAGACGGCATACGAGATAACCTCAGTGACTGGAGTTCAGACGTGTGCTCTTCCGA	
Index Primer2	CAAGCAGAAGACGGCATACGAGATTCTAAGCGTGACTGGAGTTCAGACGTGTGCTCTTCCGA	
Index Primer3	CAAGCAGAAGACGGCATACGAGATCTGTCATGTGACTGGAGTTCAGACGTGTGCTCTTCCGA	
Index Primer4	CAAGCAGAAGACGGCATACGAGATGGAGGTGGTGACTGGAGTTCAGACGTGTGCTCTTCCGA	
Index Primer5	CAAGCAGAAGACGGCATACGAGATGCTCGATGTGACTGGAGTTCAGACGTGTGCTCTTCCGA	
Index Primer6	CAAGCAGAAGACGGCATACGAGATTAGAGTAGTGACTGGAGTTCAGACGTGTGCTCTTCCGA	
Index Primer7	CAAGCAGAAGACGGCATACGAGATTCAGTCTGTGACTGGAGTTCAGACGTGTGCTCTTCCGA	
Index Primer8	CAAGCAGAAGACGGCATACGAGATTTCCAAGGTGACTGGAGTTCAGACGTGTGCTCTTCCGA	
Index Primer9	CAAGCAGAAGACGGCATACGAGATTAATCGGGTGACTGGAGTTCAGACGTGTGCTCTTCCGA	
Index Primer10	CAAGCAGAAGACGGCATACGAGATCGCTGCCGTGACTGGAGTTCAGACGTGTGCTCTTCCGA	
Index Primer11	CAAGCAGAAGACGGCATACGAGATATGATGGGTGACTGGAGTTCAGACGTGTGCTCTTCCGA	
Index Primer12	CAAGCAGAAGACGGCATACGAGATCTTGTTAGTGACTGGAGTTCAGACGTGTGCTCTTCCGA	
m923-cas9-del-F	CAGTTCCCCATAGTTGTCCTG	Detect CRISPR/Cas9 deletion in mouse
m923-cas9-del-R	GGAAGAGTTGCCTTCAGAGC	
m923.5'screenF	TCTTTAGTGCTCAGTTAACAGCT	Detect 5' loxP insertion in mouse
m923-5'LONG-lox-sphI-rev6	AGTCCTCTATCTCTTTAAAGCATGCATAAC	
m923-3'lox-F2	GTTCTAAAGCTTATAACTTCGTATAGCA	Detect 3' loxP insertion in mouse
m923-3'screen-R2	TGCCTCACCAAATTCTCACA	
m9235'screenF	TCTTTAGTGCTCAGTTAACAGCT	Genotype 923WT
m923_5'WTR	AGAGTAGTGTTCAGGATGCCT	
m923-cas9-del-F	CAGTTCCCCATAGTTGTCCTG	Genotype 923del
m923-cas9-del-R	GGAAGAGTTGCCTTCAGAGC	
m923-cas9-del-F	CAGTTCCCCATAGTTGTCCTG***	Genotype 923large
m923-cas9-del-R	GGAAGAGTTGCCTTCAGAGC	
AD1_noMX	AAT GAT ACG GCG ACC ACC GAG ATC TAC ACT CGT CGG CAG CGT CAG ATG TG	ATAC-seq Forward Primer
Ad2.l_TAAGGCGA	CAAGCAGAAGACGGCATACGAGATTCGCCTTAGTCTCGTGGGCTCGGAGATGT	ATAC-seq Index Reverse Primer
Ad2.2_CGTACTAG	CAAGCAGAAGACGGCATACGAGATCTAGTACGGTCTCGTGGGCTCGGAGATGT	ATAC-seq Index Reverse Primer
Ad2.3_AGGCAGAA	CAAGCAGAAGACGGCATACGAGATTTCTGCCTGTCTCGTGGGCTCGGAGATGT	ATAC-seq Index Reverse Primer
Ad2.4_TCCTGAGC	CAAGCAGAAGACGGCATACGAGATGCTCAGGAGTCTCGTGGGCTCGGAGATGT	ATAC-seq Index Reverse Primer
Ad2.5_GGACTCCT	CAAGCAGAAGACGGCATACGAGATAGGAGTCCGTCTCGTGGGCTCGGAGATGT	ATAC-seq Index Reverse Primer
Ad2.6_TAGGCATG	CAAGCAGAAGACGGCATACGAGATCATGCCTAGTCTCGTGGGCTCGGAGATGT	ATAC-seq Index Reverse Primer
Ad2.7_CTCTCTAC	CAAGCAGAAGACGGCATACGAGATGTAGAGAGGTCTCGTGGGCTCGGAGATGT	ATAC-seq Index Reverse Primer
Ad2.8_CAGAGAGG	CAAGCAGAAGACGGCATACGAGATCCTCTCTGGTCTCGTGGGCTCGGAGATGT	ATAC-seq Index Reverse Primer
AD2.0563_ATGTAATGG	CAAGCAGAAGACGGCATACGAGATCCATTACATGTCTCGTGGGCTCGGAGATGT	ATAC-seq Index Reverse Primer
AD2.0564_CTGTGCGTA	CAAGCAGAAGACGGCATACGAGATTACGCACAGGTCTCGTGGGCTCGGAGATGT	ATAC-seq Index Reverse Primer
AD2.0565_TGAGTAAGT	CAAGCAGAAGACGGCATACGAGATACTTACTCAGTCTCGTGGGCTCGGAGATGT	ATAC-seq Index Reverse Primer
AD2.0566_CTACCCACC	CAAGCAGAAGACGGCATACGAGATGGTGGGTAGGTCTCGTGGGCTCGGAGATGT	ATAC-seq Index Reverse Primer
AD2.0567 _GTCACACGT	CAAGCAGAAGACGGCATACGAGATACGTGTGACGTCTCGTGGGCTCGGAGATGT	ATAC-seq Index Reverse Primer
AD2.0568_ACTTTGCGT	CAAGCAGAAGACGGCATACGAGATACGCAAAGTGTCTCGTGGGCTCGGAGATGT	ATAC-seq Index Reverse Primer

Amplicon	*Sequence*	*rsID*	*C57B6*	*BALB*
Ivl	TGGGTCAGTCACTTAAGCAAGAGAAAGCTTCAAGG**A**AACAGCAGCTAGATTACTCACATCTAGAA<br>CAGGAGAAGGAGCTCTCAGACCAGCCACTGGATCAAGCACTAGTAAAGAAGGGTAAACAACTGG<br>AAAGGAAGAAACACGAATTGGAGAACCGGACACAGCAGGAGAAGTAGatagagcaattagtaccaagcact<br>gactaagccagtccaaccagtgaaaggagacg**t**gctcactacagagaagcagcagcagaa	rs32990753	A	G
		rs32990750	T	C
Lce6a	TCCAGAACACTGTCAGCCATAAGGAAATTCATCACCCACAACCTCGCTGTCTTAGGGGTAGTAC<br>CACCTACCACTGCAAAGAAGAAGAGTGCTAAgaaactgggcacaaacgagggtaaatagctacaacaacctttccaga<br>taaactcatgaatt**t**caccaggaaggccaggccctccacctctcttggctagaaaaacatttcttgtttctttctttagc**c**taccctttcagttcaac<br>aacaataaaattgatcatggtgc	rs31222976	T	C
		rs31417097	C	T

***Primer doesn’t exactly match sanger sequence..(CAATTTCCCATAGTTGTCCTG)

Supplementary Table 12. ATAC-seq library statistics. Included statistics reported as recommended by ENCODE guidelines.

Sample	*Sex*	*Total Reads pairs*	*Post filtering read pairs*	*Alignment Rate*	*Library complexity*						
					NFR	*PBC1*	*PBC2*	*NFR?*	*mono-nucleosome peak?*	*FRiP*	*TSS enrichment*
WT_1	M	44,104,640	39,024,826	79.13	0.829	0.835	6.167	✔	✔	0.036	8.214
WT_3	F	29,518,179	32,720,098	95.79	0.856	0.864	7.539	✔	✔	0.052	9.760
WT_4	F	33,796,509	36,502,990	95.37	0.831	0.831	6.434	✔	✔	0.059	11.093
Del_1	M	58,966,233	28,521,337	92.66	0.760	0.765	4.254	✔	✔	0.088	14.844
Del_2	M	43,934,578	21,294,165	96.73	0.779	0.786	4.774	✔	✔	0.026	6.913
Del_3	M	26,577,482	14,366,531	96.92	0.842	0.859	7.341	✔	✔	0.106	14.729
Del_4	M	26,880,711	14,383,001	97	0.820	0.837	6.303	✔	✔	0.092	13.897
Large_1	M	74,101,870	39,840,266	99.46	0.837	0.841	6.105	✔	✔	0.033	7.750
Large_2	M	60,641,676	39,482,578	99.43	0.962	0.967	31.580	✔	✔	0.042	9.260
Large_4	M	47,393,679	39,766,656	82	0.799	0.809	5.320	✔	✔	0.027	7.650

Reproducibility	*IDR Values*		*naïve overlap peaks*	*IDR peaks*
**Per Genotype**	**Rescue**	**self consistency**		
WT	1.1506	1.3738	169158	78032
$923^{del/del}$	1.3075	1.5896	126285	51601
$923^{large/large}$	1.2698	2.0893	190190	98605

Supplementary Table 13. Transcription Factor Motif (FIMO) hit analyses for ChIP-seq peaks for relevant ENCODE cell lines. FIMO computes a log-likelihood ratio score for each motif at each position and converts these scores to *P*-values using dynamic programming (Supplementary Reference 1).

TF	*Cell Line*	*Rep*	*Peaks*	*Ref*	*Alt*	*P value*	*FIMO Hits*	*Hits/Peak*	*ENCODE Experiment*	*Hyperlink*
MAZ	GM12878	1	23951	C	T	1.00E-05	11872	0.50	ENCSR903MVU	https://www.encodeproject.org/experiments/ENCSR903MVU/
MAZ	GM12878	2	23391	C	T	1.00E-05	11647	0.50	ENCSR000DZA	https://www.encodeproject.org/experiments/ENCSR000DZA/
MAZ	HEK293	1	42805	C	T	1.00E-05	41649	0.97	ENCSR290SSQ	https://www.encodeproject.org/experiments/ENCSR290SSQ/
MAZ	HepG2	1	27513	C	T	1.00E-05	17233	0.63	ENCSR700PNE	https://www.encodeproject.org/experiments/ENCSR700PNE/
MAZ	HepG2	2	15481	C	T	1.00E-05	8451	0.55	ENCSR000EDN	https://www.encodeproject.org/experiments/ENCSR000EDN/
MAZ	K562	1	32781	C	T	1.00E-05	15428	0.47	ENCSR163IUV	https://www.encodeproject.org/experiments/ENCSR163IUV/
MAZ	K562	2	26881	C	T	1.00E-05	13727	0.51	ENCSR643JRH	https://www.encodeproject.org/experiments/ENCSR643JRH/
ZNF263	HEK293	1	43445	A	G	1.00E-05	33451	0.77	ENCSR000EVD	https://www.encodeproject.org/experiments/ENCSR000EVD/
ZNF263	HepG2	1	27777	A	G	1.00E-05	14140	0.51	ENCSR313MMD	https://www.encodeproject.org/experiments/ENCSR313MMD/
ZNF263	K562	1	4005	A	G	1.00E-05	4856	1.21	ENCSR000EWN	https://www.encodeproject.org/experiments/ENCSR000EWN/
NFIC	GM12878	1	38795	A	G	5.00E-04	22757	0.59	ENCSR000BRN	https://www.encodeproject.org/experiments/ENCSR000BRN/
NFIC	K562	1	48550	A	G	5.00E-04	38504	0.79	ENCSR796ITY	https://www.encodeproject.org/experiments/ENCSR796ITY/
SPI1	GM12878	1	43950	G	A	5.00E-04	27129	0.62	ENCSR000BGQ	https://www.encodeproject.org/experiments/ENCSR000BGQ/
SPI1	K562	1	32589	G	A	5.00E-04	22472	0.69	ENCSR000BGW	https://www.encodeproject.org/experiments/ENCSR000BGW/

Supplementary Reference

1. Grant, C. E., Bailey, T. L. & Noble, W. S. FIMO: scanning for occurrences of a given motif. *Bioinformatics* **27**, 1017-1018, doi:10.1093/bioinformatics/btr064 (2011).
