# Supplementary material for: Selective sweep for an enhancer involucrin allele identifies skin barrier adaptation out of Africa
Source: Nat Commun. 2021 May 7;12:2557. doi: 10.1038/s41467-021-22821-w (PMC8105351; doi:10.1038/s41467-021-22821-w)
Supplement: Supplementary file 3 — Description of Additional Supplementary Files [file 41467_2021_22821_MOESM3_ESM.pdf]

Description of Additional Supplementary Files

File Name: Supplementary Data 1
Description: SNPs in clusters with high CMS scores (>2) for each population. SNPs with FDR (Benjamini-Hochberg) <0.05 are bolded and red highlighted.

File Name: Supplementary Data 2
Description: . iSAFE scores for EDC SNPs in CEU, JPT/CHB, YRI, FIN, and IBS. SNPs with iSAFE scores >0.10 are bolded and red highlighted. SNPs with 0.095<iSAFE score<0.10 are bolded and orange highlighted.

File Name: Supplementary Data 3
Description: Defined haplotypes for each 1KGP population (CEU, FIN, and IBS) based on SNPs that are in linkage disequilibrium (r^2<0.80) with SNPs that have top CMS (bold) and/or iSAFE>0.10 (pink) scores and also for SNPs with 0.095<iSAFE<0.10 (orange).

File Name: Supplementary Data 4
Description: Ranked list of differentially expressed genes between $923^{large/large}$ and WT mice whole skin from RNA-seq. List ranked by log2FC. Statistical analysis using Limma's generalized linear model moderated two-sided t-tests with 22 degrees of freedom and FDR (Benjamini-Hochberg, adj.P.val) <0.05 and logFC ≤|2| cutoffs were used.

File Name: Supplementary Data 5
Description: Ranked list of differentially expressed genes between $^{923large/+}$ and WT mice whole skin from RNA-seq. List ranked by log2FC. Statistical analysis using Limma's generalized linear model moderated two-sided t-tests with 22 degrees of freedom and FDR (Benjamini-Hochberg, adj.P.val) <0.05 and logFC ≤ |2| cutoffs were used.

File Name: Supplementary Data 6
Description: Ranked list of differentially expressed genes between Ivl-/- and WT mice whole skin from RNA-seq. List ranked by log2FC. Statistical analysis using Limma's generalized linear model moderated twosided t-tests with 22 degrees of freedom and FDR (Benjamini-Hochberg, adj.P.val) <0.05 and logFC ≤ |2| cutoffs were used.
