# Supplementary material for: Selective sweep for an enhancer involucrin allele identifies skin barrier adaptation out of Africa
Source: Nat Commun. 2021 May 7;12:2557. doi: 10.1038/s41467-021-22821-w (PMC8105351; doi:10.1038/s41467-021-22821-w)
Supplement: Supplementary file 10 — Reporting summary [file 41467_2021_22821_MOESM10_ESM.pdf]

nature research

Corresponding author(s): Cristina de Guzman Strong

Last updated by author(s): Mar 10, 2021

Reporting Summary

Nature Research wishes to improve the reproducibility of the work that we publish. This form provides structure for consistency and transparency in reporting. For further information on Nature Research policies, see our Editorial Policies and the Editorial Policy Checklist.

Statistics

For all statistical analyses, confirm that the following items are present in the figure legend, table legend, main text, or Methods section.

n/a	Confirmed	
☐	☒	The exact sample size (*n*) for each experimental group/condition, given as a discrete number and unit of measurement
☐	☒	A statement on whether measurements were taken from distinct samples or whether the same sample was measured repeatedly
☐	☒	The statistical test(s) used AND whether they are one- or two-sided<br>*Only common tests should be described solely by name; describe more complex techniques in the Methods section.*
☒	☐	A description of all covariates tested
☐	☒	A description of any assumptions or corrections, such as tests of normality and adjustment for multiple comparisons
☐	☒	A full description of the statistical parameters including central tendency (e.g. means) or other basic estimates (e.g. regression coefficient) AND variation (e.g. standard deviation) or associated estimates of uncertainty (e.g. confidence intervals)
☐	☒	For null hypothesis testing, the test statistic (e.g. F, t, r) with confidence intervals, effect sizes, degrees of freedom and P value noted<br>*Give P values as exact values whenever suitable.*
☒	☐	For Bayesian analysis, information on the choice of priors and Markov chain Monte Carlo settings
☒	☐	For hierarchical and complex designs, identification of the appropriate level for tests and full reporting of outcomes
☐	☒	Estimates of effect sizes (e.g. Cohen's d, Pearson's r), indicating how they were calculated

Our web collection on statistics for biologists contains articles on many of the points above.

Software and code

Policy information about availability of computer code

Data collection	This section applies to the allele-specific ChIP-seq analysis for the ENCODE or other publicly available genomic datasets.<br>Allele-specific ChIP-seq analysis (Extracted from Methods section): All ChIP-Seq data, including IDR thresholded peaks bed and bam alignment files were downloaded from ENCODE (accession codes in Data Availability section, links provided in Table S19). Variant calls in vcf format were downloaded from ENCODE (HepG2: ENCSR319QHO, K562: ENCSR053AXS), the Platinum Genomes project70 (https://github.com/Illumina/PlatinumGenomes), and the HEK293 genome project (http://hek293genome.org/v2/)71 and if necessary converted to hg38 using liftOver using the UCSC genome browswer portal. Variants were further filtered to only include heterozygous variants.
Data analysis	The following details provide information for software and packages and their versions that were used for the analyses. References or links are provided where possible. No custom code was used given the software and packages.<br><br>Basecalls and demultiplexing were performed with Illumina's bcl2fastq software v2.20 with a maximum of one mismatch in the indexing read. RNA-seq reads were then aligned to the Ensembl release 96 top-level assembly with STAR version 2.0.4b. Gene counts were derived from the ATnumber of uniquely aligned unambiguous reads by Subread:featureCount version 1.4.5. Sequencing performance was assessed for the total number of aligned reads, total number of uniquely aligned reads, and features detected. The ribosomal fraction, known junction saturation, and read distribution over known gene models were quantified with RSeQC version 2.3. All gene counts were then imported into the R/Bioconductor package EdgeR version 3.22.0 and TMM normalization size factors were calculated to adjust for samples for differences in library size. Ribosomal genes and genes not expressed in the smallest group size minus one samples greater than one count-per-million were excluded from further analysis. The TMM size factors and the matrix of counts were then imported into the R/Bioconductor package Limma version 3.36.5. Performance of the samples was assessed with Spearman correlations, and a Multi-Dimensional Scaling plot, and hierarchical clustering. Weighted likelihoods based on the observed mean-variance relationship of every gene and sample were then calculated for all samples with the voomWithQualityWeights. The performance of all genes was assessed with plots of the residual standard deviation of every gene to their average log-count with a robustly fitted trend line of the residuals. Differential expression analysis was then performed to analyze for differences between conditions and the results were filtered for only those genes with Benjamini-Hochberg false-discovery rate

adjusted p-values less than or equal to 0.05, and a log2(fold change) > |2|.The R/Bioconductor package heatmap3 version1.1.6 and Pathview version 1.18.2 was used to display heatmaps or annotated KEGG graphs across groups of samples for each GO term or KEGG pathway (respectively) with a Benjamini-Hochberg false-discovery rate adjusted p-value less than or equal to 0.05.

Allele-specific gene expression (extracted from Methods section): De-multiplexed reads were mapped using Bowtie2 version 2.3.5.1 and visualized using the IGV viewer. The proportion of nucleotides at each informative SNP in the amplicon was calculated by IGV.

ATAC-seq (extracted from Methods section): ATAC-seq data was processed using the ENCODE ATAC-seq processing pipeline using Caper with Conda (https://github.com/ENCODE-DCC/atac-seq-pipeline)(ref. 66). Reads were mapped using Bowtie2 version 2.3.5.1, and filtered to remove unmapped reads, duplicates, and reads mapping to chrM. Peaks were called on each replicate using MACS267 . Biological replicates were included if both the rescue and self-consistency IDR values per genotype were below (or very near) 2 (Table S18). Differential accessibility was assessed using EdgeR68 within the DiffBind R package version 2.12.0 (http://bioconductor.org/packages/DiffBind/)(FDR <0.5, log2(FC) >|2|).

Transcription Factor Binding Predictions (extracted from Methods section): Both reference and alternate nucleotides for a given SNP were rigorously queried and centered at position 25 in a 50 bp window were analyzed with PROMO 3.0 (refs 71,72) and ConSite (http://consite.genereg.net/cgi-bin/consite). PROMO 3.0 with TRANSFAC version 8.3 considered only human sites and human factors with 15% maximum matrix dissimilarity rate and Consite utilized the option for all transcription factors profiles with a minimum specificity of 10 bits and transcription factor score cutoff of 80% in a single sequence. JASPAR73 with a relative profile score threshold 80% was used to analyze each SNP allele (reference and alternate independently) centered at position 15 in a 30 bp window. HAPLOREG v.474 was queried for a given SNP rsID.

Allele-specific ChIP-seq analysis (extracted from Methods section): Sequences for each ChIP-seq peak were extracted using bedtools and the hg38 UCSC genome reference. Motifs for each TF from JASPAR 202073 (MAZ: MA1522.1, NFIC: MA0161.1, SPI1: MA0080.1, ZNF263: MA0528.2) and used FIMO (part of the MEME suite v5.3.3) (ref. 77) were downloaded to search for occurrences of each motif with --max-stored-scores set to 1E-8. Since the appropriate p-value threshold is dependent on the motif length and information content, p-values were scanned for each motif search across the values 1E-5, 5E-4, 1E-4, 5E-3, and 1E-3. For each motif, the p-value was set based on whether there was an average of 0.5-1 motifs found per ChIP-seq peak across each of the datasets for a given TF (Table S19). Bedtools (v2.26.0, https://bedtools.readthedocs.io/) was used to overlap identified motifs with heterozygous SNPs using the variant calls (.vcf file) from the appropriate cell line. Samtools (v1.3.1, http://www.htslib.org/) view with the -L flag was used to next extract all alignments that overlapped identified ChIP-seq peaks. Aligned reads were overlapped to identify each SNP, manually curated to ensure the SNP corresponded to the queried variant, and counted for the number of reads supporting each base. Further, only SNPs with coverage >= 5 and within 50 bp of the peak center were retained. Results from IRF1 and RELA are not included since no SNPs met these criteria.

For manuscripts utilizing custom algorithms or software that are central to the research but not yet described in published literature, software must be made available to editors and reviewers. We strongly encourage code deposition in a community repository (e.g. GitHub). See the Nature Research guidelines for submitting code & software for further information.

Data

Policy information about availability of data

All manuscripts must include a data availability statement. This statement should provide the following information, where applicable:

- Accession codes, unique identifiers, or web links for publicly available datasets
- A list of figures that have associated raw data
- A description of any restrictions on data availability

All data supporting the findings of this study are available within this article and the supplemental material. Raw RNA and ATAC sequencing data are available in NCBI Gene Expression Omnibus (GEO) using the accession code GSE158870 [https://www.ncbi.nlm.nih.gov/geo/query/acc.cgi?acc=GSE158870]. The following ENCODE ChIP datasets were used: GM12878-MAZ (ENCSR903MVU, ENCSR000DZA), HEK293-MAZ (ENCSR290SSQ), HepG2-MAZ (ENCSR700PNE, ENCSR000EDN), K562-MAZ (ENCSR163IUV, ENCSR643JRH), HEK293-ZNF263 (ENCSR000EVD), HepG2-ZNF263 (ENCSR313MMD), K562-ZNF263 (ENCSR000EWN), GM12878-NFIC (ENCSR000BRN), K562-NFIC (ENCSR796ITY), GM12878-SPI1 (ENCSR000BGQ), and K562-SPI1 (ENCSR000BGW). Links to the ENCODE datasets and the reporting summary are available in the supplemental material. Source data are also provided with this paper.

Field-specific reporting

Please select the one below that is the best fit for your research. If you are not sure, read the appropriate sections before making your selection.

☒ Life sciences ☐ Behavioural & social sciences ☐ Ecological, evolutionary & environmental sciences

For a reference copy of the document with all sections, see nature.com/documents/nr-reporting-summary-flat.pdf

Life sciences study design

All studies must disclose on these points even when the disclosure is negative.

Sample size	Sample sizes for the mice were chosen based on age-, littermate-, gender-matched (as much as possible) and weight-matched criteria. Sample sizes of 3 or 4 per genotype and assay were sufficient given the feasibility of meeting the 4 criteria mentioned above for a given litter.
Data exclusions	Biological replicates for ATAC-seq were excluded if it did not meet both the rescue and self-consistency IDR values per genotype below (or very near) 2 (Table S18).
Replication	We verified the RNA-seq findings for decreased IVL and increased Lce6a expressions using qPCR. Prior to ATAC-sequencing, all samples exhibited the expected periodicity of insert length and were enriched for reads at transcription start sites demonstrating uniform processing of the samples. Furthermore, we followed the ENCODE guidelines to rigorously analyze the ATAC-seq data. These measures allowed us to determine that the DARs were reproducible across the biological replicates (Figure S6).We repeated one WT and one 923large ATAC-seq library each and replicated the findings.
Randomization	Mice were allocated based on genotype.
Blinding	Investigators were blinded at data collection. They were not blinded for RNA-seq and ATAC-seq data analyses so that comparisons between the group categories can be appropriately performed. For all other experimental analyses, investigators were blinded.

Reporting for specific materials, systems and methods

We require information from authors about some types of materials, experimental systems and methods used in many studies. Here, indicate whether each material, system or method listed is relevant to your study. If you are not sure if a list item applies to your research, read the appropriate section before selecting a response.

Materials & experimental systems

n/a	Involved in the study
☐	☒ Antibodies
☐	☒ Eukaryotic cell lines
☒	☐ Palaeontology and archaeology
☐	☒ Animals and other organisms
☐	☒ Human research participants
☒	☐ Clinical data
☒	☐ Dual use research of concern

Methods

n/a	Involved in the study
☒	☐ ChIP-seq
☒	☐ Flow cytometry
☒	☐ MRI-based neuroimaging

Antibodies

Antibodies used	The following antibodies were used for immunofluorescence: rabbit IVL (4b-KSCN, 1:200) and chicken K14 (5560, 1:500) custom antibodies (courtesy of J. Segre), goat anti-rabbit (Alexa Fluor 488 #A-11008, 1:500), and goat anti-chicken (Alexa Fluor 594 #A11042, 1:500) IgG antibodies (Life Technologies, Frederick, MD).
Validation	Each primary antibody has been validated using Western blots that verify appropriate migration of the expected kDa band prior to immunofluorescent experimental staining (Rabbit IVL, chicken K14). Secondary antibodies were purchased from Life Technologies, Frederick, MD that meet quality control prior to commercial purchase.

Eukaryotic cell lines

Policy information about cell lines

Cell line source(s)	SP-1 mouse keratinocyte cell line were originally derived from SENCAR mice and kindly provided by Dr. Stuary Yuspa (NCI).
Authentication	SP-1 cells were not authenticated but undergo low number of passages.
Mycoplasma contamination	SP-1 cells were not tested for mycoplasma.
Commonly misidentified lines (See ICLAC register)	No commonly misidentified cell lines were used in this study.

Animals and other organisms

Policy information about studies involving animals; ARRIVE guidelines recommended for reporting animal research

Laboratory animals	Mus musculus, C57BL/6 and BALV/cBYJ strains, ages embryos -4 months of age, males and females. All mice were group-housed in

	cages with bedding and nesting material in pathogen-free, barrier facilities (65-75 degrees C and 40-60% humidity) with a 12 hour light/dark cycle at Washington University School of Medicine (St. Louis, MO).
Wild animals	The study did not involve wild animals.
Field-collected samples	The study did not involve samples collected from the field.
Ethics oversight	The Division of Comparative Medicine Animal Studies Committee at Washington University in St. Louis School of Medicine approved the animal study protocol.

Note that full information on the approval of the study protocol must also be provided in the manuscript.

Human research participants

Policy information about studies involving human research participants

Population characteristics	There are no human research participants that were examined in this study. Human population genetic data was obtained from publicly available datasets (lKGP).
Recruitment	There are no human research participants that were examined in this study. Patients for the cloned human alleles were recruited from previous publication with no bias that would have an affect on the reported study here.
Ethics oversight	Washington University in St. Louis School of Medicine

Note that full information on the approval of the study protocol must also be provided in the manuscript.

April 2020
